# Skipping of Exon 20 in *EP300*: A Novel Variant Linked to Rubinstein–Taybi Syndrome With Atypical and Severe Clinical Manifestations

**DOI:** 10.1111/cge.14654

**Published:** 2024-11-27

**Authors:** Lisa Pavinato, Silvia Carestiato, Slavica Trajkova, Lorena Sorasio, Giovanna Mantovani, Luisa De Sanctis, Jennifer Kerkhof, Haley McConkey, Jessica Rzasa, Emily Todd, Maria Balzo, Simona Cardaropoli, Alessandro Bruselles, Silvia De Rubeis, Joseph D. Buxbaum, Marco Tartaglia, Bekim Sadikovic, Giovanni Battista Ferrero, Alfredo Brusco

**Affiliations:** ^1^ Institute for Oncology Research, BIOS+ Bellinzona Switzerland; ^2^ Università Della Svizzera Italiana Lugano Switzerland; ^3^ Department of Neurosciences Rita Levi‐Montalcini University of Turin Turin Italy; ^4^ Medical Genetics Unit, Città Della Salute e Della Scienza University Hospital Turin Italy; ^5^ Santa Croce e Carle Hospital, Pediatrics Unit Cuneo Italy; ^6^ Department of Clinical Sciences and Community Health University of Milan Milano Italy; ^7^ Endocrinology Unit, Fondazione IRCCS ca’ Granda Ospedale Maggiore Policlinico Milan Italy; ^8^ Department of Public Health and Pediatric Sciences University of Turin Turin Italy; ^9^ Verspeeten Clinical Genome Centre, London Health Sciences Centre London Ontario Canada; ^10^ Department of Pathology and Laboratory Medicine Western University London Ontario Canada; ^11^ UCHealth Adult Genetics Clinic–Anschutz Medical Campus, Adult Medical Genetics Clinic Aurora USA; ^12^ Department of Oncology and Molecular Medicine Istituto Superiore di Sanità Roma Italy; ^13^ Seaver Autism Center for Research and Treatment, Icahn School of Medicine at Mount Sinai New York New York USA; ^14^ Department of Psychiatry Icahn School of Medicine at Mount Sinai New York New York USA; ^15^ The Mindich Child Health and Development Institute, Icahn School of Medicine at Mount Sinai New York New York USA; ^16^ Friedman Brain Institute, Icahn School of Medicine at Mount Sinai New York New York USA; ^17^ Department of Neuroscience Icahn School of Medicine at Mount Sinai New York New York USA; ^18^ Molecular Genetics and Functional Genomics, Bambino Gesù Children's Hospital, IRCCS Rome Italy; ^19^ University of Torino Department of Clinical and Biological Sciences Orbassano Italy; ^20^ Medical Genetics Unit and Thalassemia Center, San Luigi University Hospital Orbassano Italy

**Keywords:** *EP300*, episignature, *GNAS*, p300 ring domain, Rubinstein–Taybi

## Abstract

Rubinstein–Taybi syndrome (RSTS) is a rare autosomal dominant neurodevelopmental disorder linked to haploinsufficiency of *CREBBP* (RSTS1) and *EP300* (RSTS2) genes. Characteristic features often include distinctive facial traits, broad thumbs and toes, short stature, and various degrees of intellectual disability. The clinical presentation of RSTS is notably variable, making it challenging to establish a clear genotype–phenotype correlation, except for specific variants which cause the allelic Menke–Hennekam syndrome. Trio exome analysis, data collection via networking and GeneMatcher platforms, transcript processing analysis, and DNA methylation profiling were performed. We identified two unrelated patients with de novo variants in *EP300* (NM_001429.4: c.3671+5G>C; c.3671+5_3671+8delGTAA) predicted to cause in‐frame exon 20 skipping, confirmed in one patient. In silico 3D protein modeling suggested that exon 20 deletion (comprising 27 amino acids) likely alters the structural conformation between the RING_CBP‐p300 and HAT‐KAT11 domains. Clinically, both patients displayed severe RSTS2‐like clinical features, including autism spectrum disorder, speech delay, hearing loss, microcephaly, developmental delay, and intellectual disability, alongside ocular, respiratory, and cardiovascular abnormalities. Additionally, one patient developed early‐onset colorectal cancer. DNA methylation profiling in Subject #1 confirmed RSTS but did not align with the specific episignatures for RSTS1 or RSTS2. We propose that skipping of exon 20 in *EP300* is associated with a distinct form of Rubinstein–Taybi syndrome featuring clinical characteristics not fully aligning with RSTS1 or RSTS2. Our findings increase the understanding of RSTS genetic and molecular basis and stress the need for further research to establish definitive genotype–phenotype correlations.

## Graphical abstract

We propose that variants leading to the skipping of exon 20 in the *EP300* gene result in a severe form of Rubinstein–Taybi syndrome (RSTS). In our study, we identified two patients with de novo variants, and in one case, we confirmed exon 20 skipping. DNA methylation analysis supported the RSTS diagnosis; however, the episignature did not align with the known RSTS1 or RSTS2 profiles, suggesting a distinct subtype of RSTS.

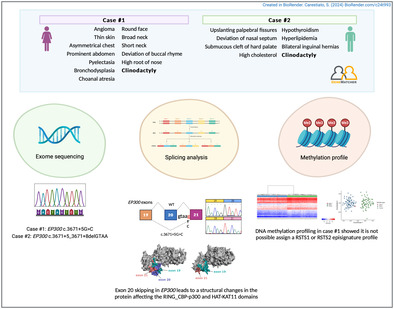

## 1 Introduction

Rubinstein–Taybi syndrome (RSTS) is a rare autosomal dominant neurodevelopmental disorder divided into two main types: Type 1 (RSTS1; OMIM #180849) and Type 2 (RSTS2; OMIM #613684), which respectively result from mutations in the paralogous genes *CREBBP* and *EP300* [1, 2]. RSTS1 typically presents more severe symptoms, including distinctive facial features (such as a broad nose and arched eyebrows), broad thumbs and toes, intellectual disability (ID), and growth retardation, often with cardiac and ophthalmologic issues. Though both types share several features, RSTS2 usually exhibits less severe skeletal abnormalities, milder facial characteristics, and generally mitigated ID and growth issues [3].

Variants associated with RSTS involve more than 500 pathogenic variants in *CREBBP* and over 100 in *EP300*, lacking a clear genotype–phenotype correlation [3]. Complicating this phenotypic landscape, mutations in the terminal exons of these genes are linked to Menke–Hennekam syndrome (MKHK1; OMIM #618332), an allelic disorder to RSTS, marked by variable ID and syndromic traits. Variants associated with RSTS and MHS have distinct DNA methylation signatures, or episignatures, illustrating their differential functional impacts [4, 5].

This report discusses two unrelated cases featuring intronic variants at the acceptor splice site of *EP300* intron 20, causing in‐frame exon 20 exclusion. Analysis of these cases suggests a specific genotype–phenotype correlation linked to variants affecting the functional domain encoded by exon 20.

## 2 Material and Methods

### 2.1 Genetic Analyses

Genetic analyses included array‐CGH and exome sequencing/gene panel analysis detailed in Supporting Information. Analysis of *EP300* exon 20 splicing was performed on cDNA from blood of Subject #1 (Supporting Information). DNAm was conducted using the clinically validated EpiSign assay, following previously established methods where the DNAm data for this sample were compared to the Episign Knowledge Databases (EKD) using the Support Vector Machine (SVM)–based classification algorithm as described in supplemental file.

## 3 Results

### 3.1 Clinical Description of Cases

**FIGURE 1:**
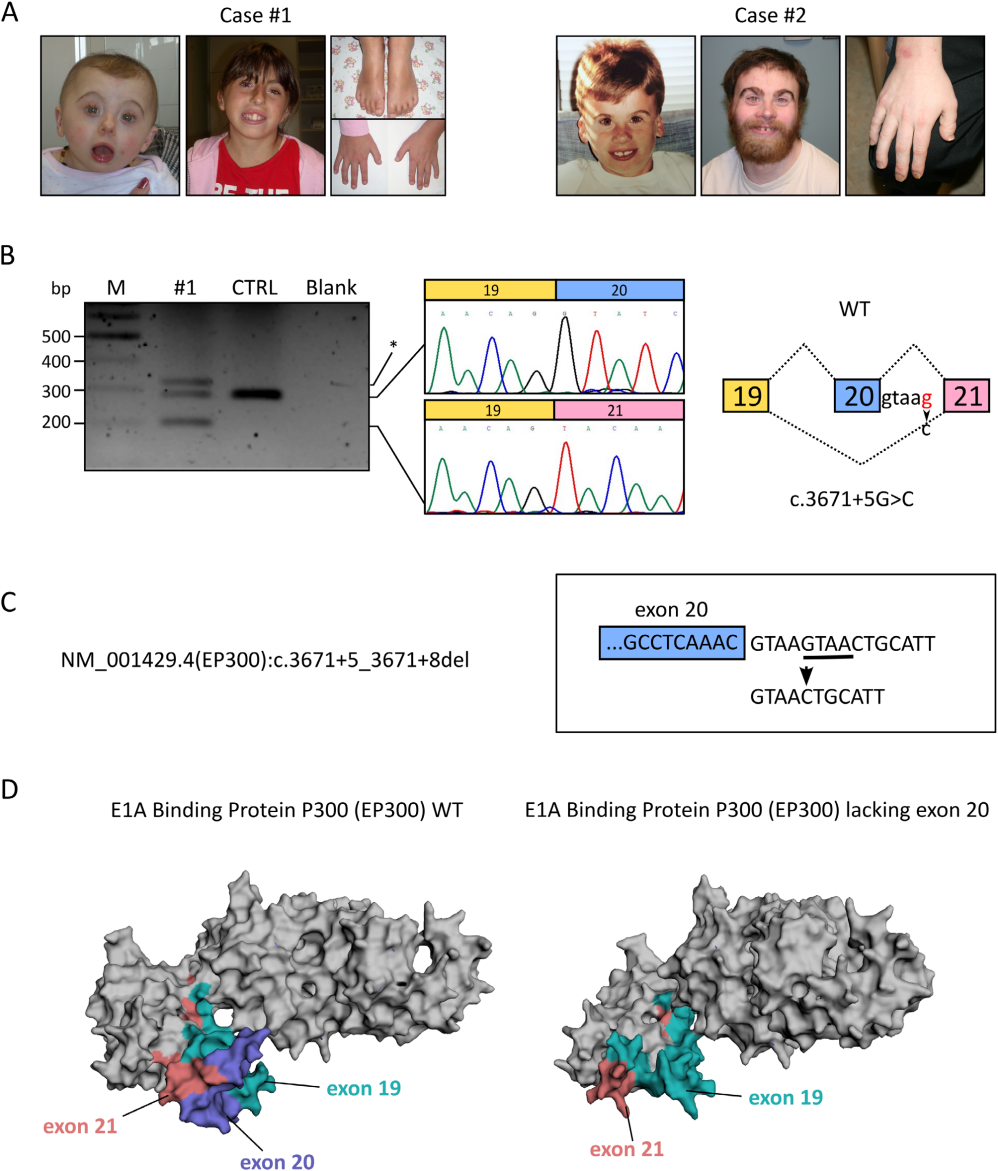
Patients and impact of the *EP300* c.3671+5G>C variant. (A) Photographs illustrate the facial features typical of Subject #1 (left) and Subject #2 (right) at different ages. The images highlight limb features such as small hands and short toes. Both patients exhibit clinodactyly, not previously reported in literature for *EP300* cases. (B) *EP300* transcript analysis using cDNA extracted from blood. RT‐PCR amplification targeting exons 19 through 21 revealed two bands. The band at approximately 300 bp corresponds to the wild‐type transcript, found also in the control sample. The band around 200 bp indicates exon 20 skipping, as confirmed by gel extraction and Sanger sequencing. An additional band, marked by an asterisk, is likely a heteroduplex. The schematic on the right illustrates these splicing events. (C) The bioinformatics prediction of the NM_001429.4(*EP300*):c.3671+5_3671+8del variant in Subject #2 shows alteration in the splicing pattern. This four‐base‐pair deletion also induces a c.3671+5G>C change, likely impacting the splicing mechanism similarly. (D) A 3D reconstruction of the EP300 protein, generated through the Protein Homology/analogY Recognition Engine (PHYRE) version 2 (Supporting Information). The reconstruction illustrates the impact of the lack of exon 20. The absence of this exon results in the loss of amino acids within the RING domain, which encompasses exons 19, 20, and 21, subsequently altering the overall structure and function of the *EP300* protein.

Subject #1 is a 9‐year‐old female who was born at full term with normal prenatal ultrasounds but exhibited minor facial abnormalities and heart defects at birth, requiring corrective surgery. She experienced developmental delays, achieving autonomous walking at 24 months and speaking at 5–6 years, with current communication skills involving simple sentences. She faced challenges like bilateral mixed hearing loss, attention issues, and self‐regulation difficulties, alongside normal sleep–wake rhythms and appropriate expressions. At 8 years old, her physical growth showed microcephaly, and echocardiography suggested possible dilation of the aortic bulb (Figure 1A and Supporting Information).

Subject #2, a 32‐year‐old male diagnosed with autism and ID, exhibited microcephaly and multiple congenital anomalies. He was mostly nonverbal, experienced anxiety and seizures, and shared some clinical features with Subject #1, such as aortic arch anomalies and hearing loss. Medical conditions included gastrointestinal issues, hypothyroidism, and frequent pneumonia. He died from a colon carcinoid tumor at 32 years of age, with a family history of late‐onset cancer on the maternal side (Supporting Information).

### 3.2 Molecular Findings

Genetic testing for Subject #1 involved extensive analysis, including karyotyping and sequencing of specific genes. Despite clinical suspicion of CHARGE syndrome, no pathogenic variants were found in related genes. However, trio exome sequencing identified a de novo pathogenic splice site variant in the *EP300* gene (NM_001429.4:c.3671+5G>C; p.?), resulting in exon 20 skipping and structural alterations to the EP300 protein (Figure 1B). For Subject #2, similar genetic tests were conducted and found the NM_001429.4:c.3671+5_3671+8delGTAA; p.? de novo splice site variant in the *EP300* gene also affecting exon 20 (Figure 1C). Both variants, absent in population databases, were classified as pathogenic according to ACMG criteria. To understand the impact of the loss of exon 20 on the EP300 protein, an in silico model was generated (Supporting Information). This model demonstrated a significant alteration in the three‐dimensional structure with a partial loss of the RING_CBP‐p300 domain (Figure 1D).

### 3.3 Variants Impacting *EP300* Exon 20 Are Not Associated With RSTS2‐Specific Episignatures

A specific DNAm profile has been reported in patients with RSTS1 and RSTS2, which are associated with variants in *CREBBP* and *EP300* [5]. Taking advantage of a blood‐derived DNA sample available for Subject #1, we performed methylation profiling, to further characterize the functional impact of the identified variant.

**FIGURE 2:**
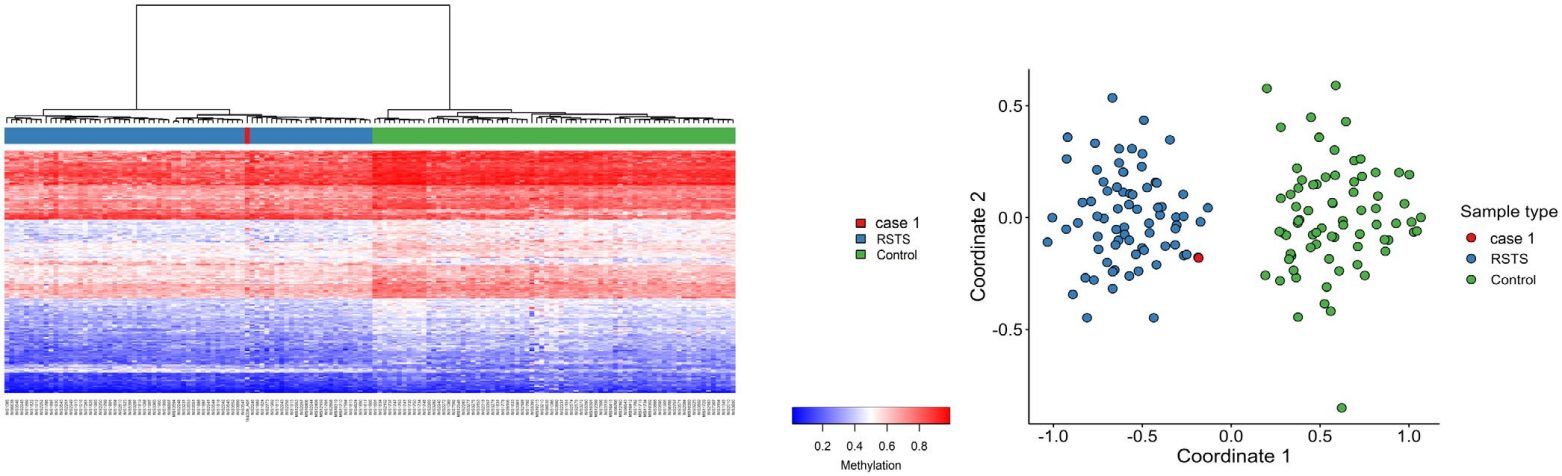
Episignature analysis of Subject 1 for Rubinstein–Taybi syndrome subtypes, RSTS1 and RSTS2. Euclidean hierarchical clustering (heatmap); right‐multidimensional scaling plot (MDS) presenting Rubinstein–Taybi syndrome samples (RSTS1 and RSTS2) in blue, controls in green, and Subject #1 in red.

The sample was compared with the DNAm profiles from a cohort of subjects clinically and molecularly diagnosed with RSTS1 and RSTS2. The analysis revealed that Subject #1 clustered with RSTS patients (Figure 2). However, the combined analysis showed it was not possible to clearly distinguish between RSTS1 or RSTS2: Unsupervised clustering was suggestive of RSTS1; however, the MVP score could not discriminate between RSTS1 and RSTS2 (0.005 vs. 0.012). To exclude the possibility of occurrence of any coding variants in *CREBBP*, we reanalyzed the exome sequencing data, but we did not find any variant in this gene.

The DNAm analysis also unexpectedly identified hypomethylation at the maternal *GNAS* locus (OMIM *139320) detailed in supplemental file and Figures S1 and S2.

### 3.4 Facial Features of *EP300* Exon 20 Skipping

To verify whether the facial features observed in our patients overlapped with previously reported cases, we compared their pictures with published images of patients affected by RSTS1 and RSTS2 using deep face gestalt analysis [6]. The results revealed that our patients did not cluster with either the RSTS1 or RSTS2 groups (Figure 3S).

Furthermore, clinical comparative analysis of different features in published patients with RSTS2 (n.52) and Subjects #1 and #2 showed differences between these two groups further highlighting unique presentations in individual case studies (Tables S1 and S2).

## 4 Discussion

We identified two patients with a splicing variant in the *EP300* gene, specifically at the donor splice site of intron 20, leading to the in‐frame exclusion of exon 20. This exon encodes 27 amino acids integral to the RING domain, crucial for regulating the histone acetyltransferase (HAT) domain [7, 8]. Variants within this domain are known to increase EP300 acetyltransferase activity and p53 acetylation, potentially contributing to cancer in some RSTS2 patients [8]. Patient #2 succumbed to a colon carcinoid tumor at age 32 years, possibly linked to this *EP300* mutation, highlighting the importance of vigilant monitoring for cancer risks among individuals with RING domain variants.

A detailed examination of our patients reveals some shared and unique features compared to 89 documented cases in the RSTS2 literature. Our patients manifested severe neurological, metabolic, and multisystem anomalies, including cardiovascular, ocular, respiratory, and skeletal issues. Features such as speech delays and intellectual disabilities (mild in one patient and severe in the other) were consistent with prior findings, although certain dysmorphic characteristics like long eyelashes and specific nasal and ear abnormalities were absent. Notably, both patients exhibited clinodactyly, a rarer characteristic among broader features like broad thumbs and halluces (Tables S1 and S2).

Our findings suggest that the skipping of exon 20 in *EP300* might result in a phenotype more aligned with RSTS1 than RSTS2, albeit without a definitive DNAm signature match. Interestingly, a distinct methylation pattern at the *GNAS* locus in Patient #1 aligns with pseudohypoparathyroidism Type 1B, confirmed by additional analyses, suggesting overlapping genetic conditions complicating the clinical and DNA methylation profiles [9].

Identifying further cases with this *EP300* variant will be critical to validate the proposed correlation with distinctive clinical features. The ClinVar database lists four cases with a similar *EP300* variant, though family contact was not feasible. The proximity of this variant to the one in our study suggests similar impacts on exon 20 splicing.

This research underscores the necessity for comprehensive genetic screening in patients with complex Rubinstein–Taybi syndrome (RSTS) phenotypes, potentially uncovering novel variants in *CREBBP* and *EP300*. Such an approach enables early, proactive management and informed decisions, potentially mitigating the disease's impact. Ultimately, this study contributes to the understanding of Rubinstein–Taybi syndrome and emphasizes the importance of acknowledging genetic complexity and phenotypic variability for accurate diagnosis and management.

## Author Contributions

Conceptualization: L.P., S.C., and S.T. Data collection and analysis: L.S., L.D.S., and E.T. Data curation: L.P., S.C., S.T., G.M., J.K., H., J.R., M.B., S.C., A.B., S.D.R., J.B., M.T., and B.S. Investigation: L.P., S.C., S.T., L.S., G.M., L.D.S., J.K., H., J.R., E.T., M.B., S.C., A.B., S.D.R., J.B., M.T., B.S., G.B.F., and A.B. Supervision: A.B. Writing – original draft: L.P., S.C., and S.T. Writing – review and editing: All authors.

## Conflicts of Interest

B.S. is a shareholder in EpiSign Inc., a company involved in commercialization of EpiSignTM technology.

### Peer Review

The peer review history for this article is available at https://www.webofscience.com/api/gateway/wos/peer‐review/10.1111/cge.14654.

## Supporting information


Data S1.

## Data Availability

The data that support the findings of this study are available from the corresponding author upon reasonable request.
