# Supplementary material for: Skipping of Exon 20 in EP300: A Novel Variant Linked to Rubinstein–Taybi Syndrome With Atypical and Severe Clinical Manifestations
Source: Clin Genet. 2024 Nov 27;107(3):354–8. doi: 10.1111/cge.14654 (PMC11790522; doi:10.1111/cge.14654)
Supplement: Supplementary file 1 — Data S1. [file CGE-107-354-s001.docx]

**Materials and methods**

*Ethics statements*

Informed consent was obtained from participating families and the study protocol was approved by the internal Ethics Committee of University of Turin (n. 0060884) according to the Declaration of Helsinki.

*Exome sequencing, prioritization, and variant calling*

DNA was extracted from total blood using the ReliaPrep Blood gDNA Miniprep kit (Promega, Madison) following manufacturer's protocol and quantified with a NanoDrop spectrophotometer (ThermoFisher Scientifics). Array-CGH was performed using a 60 K whole-genome oligonucleotide microarray (Agilent Technologies).

Patient #1 was enrolled in the Autism Sequencing Consortium (ASC) (<https://asc.broadinstitute.org/>) project and her gDNA sample was sequenced at the Broad Institute on Illumina HiSeq sequencers as previously described.^1-3^ Patient #2 was analysed using a genetic panel.

ES raw data of the trio were processed and analyzed using an in-house implemented pipeline previously described which is based on the GATK Best Practices.^4,5^ The UCSC GRCh37/hg19 version of genome assembly was used as a reference for reads alignment by means of BWA-MEM tool and the subsequent variant calling with HaplotypeCaller (GATK v3.7).^6^ We used SnpEff v.4.^7^ and dbNSFP v.3.5^8^ tools for variants functional annotation, including Combined Annotation Dependent Depletion (CADD) v.1.3,^9^ Mendelian Clinically Applicable Pathogenicity (M-CAP) v.1.0 ^10^ and Intervar v.0.1.6 for functional impact prediction.^11^ Thereby, the analysis was narrowed to variants affecting coding sequences or splice site regions. Moreover, high-quality variants were filtered against public databases (dbSNP150 and gnomAD ver.2.0.1) so that only variants with unknown frequency or having MAF <0.1%, as well as variants occurring with frequency < 1% in our population-matched database (~2000 exomes) were considered.

A further variant stratification in conformity with the American College of Medical Genetics and Genomics (ACMG) guideline,^11^ also considering mode of inheritance and functional *in silico* prediction of impact, allowed us to consider the final set of variants for possible associations with the phenotype. All variants are referred to GRCh37 annotation.

*MLPA GNAS locus analysis (MRC Holland website)*

The GNAS locus is a complex imprinted locus on chromosome 20 that generates multiple transcripts through the use of several alternative first exons that splice into a common set of downstream exons (see Figure 1S). GNAS itself encodes the Gsα protein, which is the α-subunit in the heterotrimeric G protein. Due to differential methylation of their promoters, most gene products originate from one parental allele. Transcripts GNASXL, which encodes XLαs, GNAS A/B (also referred to as 1A) and the antisense transcript GNAS-AS1 (also referred to as NESPAS) are transcribed from the paternal allele, while NESP55 (also referred to as NESP or GNAS transcript variant 4) is transcribed from the maternal allele.^12^ The most downstream promoter (GNAS exon 1) is not differentially methylated, which results in GNAS expression from both alleles in most tissues but its expression is silenced from the paternal allele in a small number of tissues.^12^

The Gsα and XLαs transcripts are involved in downstream signalling from parathyroid hormone (PTH), parathyroid hormone related protein (PTHrP) receptors and other hormone receptors like TSHR and GHRHR. The GNAS A/B transcript and the antisense transcript GNAS-AS1 are not translated into proteins but are thought to influence Gsα expression via mechanisms that remain to be determined. The STX16 gene, lastly, is a long-range control element of methylation at the GNAS locus, located more than 220 kb centromeric of GNAS.^12^

PHPIb is caused by loss-of-methylation (LOM) at GNAS A/B located within DMR 1 in the GNAS complex locus (Figure 1S). LOM can also be observed at GNAS-AS1 and GNASXL, which can also be associated with a gain-of-methylation (GOM) at NESP55. The autosomal dominant form of PHPIb can be caused by maternal heterozygous deletions in STX16.^12^

More information is available at <https://www.ncbi.nlm.nih.gov/books/NBK459117/>

*Splicing analysis*

Total RNA was extracted from peripheral blood mononuclear cells (PBMCs) using the Direct-Zol RNA MiniPrep system (Zymo Research, Irvine, CA, USA); genomic DNA was removed by treatment with DNase I (Sigma-Aldrich), following the manufacturer’s protocol. Complementary DNA (cDNA) was generated using the M-MLV Reverse Transcriptase kit (Invitrogen, Thermo Fisher Scientific). Primers for the variant EP300 (NM_001429.4) c.3671+5G>C were designed on the sequence from exon 18 to exon 23 using Ensembl Genome Browser (www.ensembl.org) and Primer3plus platform: 5’-CACAGACACTGTGTTGCTACGG Forward; 5’- CCAGATGATCTCATGGTGAAGG Reverse. cDNA was amplified with touchdown PCR using KAPA Taq PCR Kit (Roche Diagnostics, Basel Switzerland). Amplimers were visualized into 2% TBE-agarose gel using ChemiDoc Imaging System (BioRad, Hercules, CA, USA). Bands were gel-excised, and the DNA was extracted by GenElute Gel Extraction Kit (Sigma-Aldrich). Fragments were sequenced by Sanger method.

*In silico modelling*

EP300 protein was modelled using Protein Homology/analogY Recognition Engine (PHYRE) version 2,^13^ choosing intensive modelling mode option. A .pdb file was generated for both wild-type and mutant protein lacking exon 20 and imported in EzMol ^14^ interface server (ver.2.1); the colour code was generated from EzMol to indicate the aminoacid stretches encoded by exons 19 (light green), 20 (purple) and 21 (pink). The resulting image was used to visualize the 3D protein conformation.

*DNA methylation analysis by EpiSign*

DNAm was conducted using the clinically validated EpiSign assay, following previously established methods where the DNAm data for this sample was compared to the Episign Knowledge Databases (EKD) using the Support Vector Machine (SVM) based classification algorithm as previously described.^15-17^ EKD includes thousands of clinical peripheral blood DNAm profiles from disorder-specific reference and normal controls (general population samples with various age and racial backgrounds). Methylated and unmethylated signal intensities generated from the EPIC array were imported into R 4.2.1 for normalization, background correction, and filtering. Beta values were then calculated as a measure of methylation level, ranging from 0 (no methylation) to 1 (complete methylation), and processed through the established SVM classification algorithm for EpiSign disorders. The SVM decision values were converted to Methylation Variant Pathogenicity (MVP) scores ranging from 0 to 1, using the Platt scaling method. MVP scores indicate the prediction confidence for the specific episignature. Scores greater than 0.01 undergo a secondary review using hierarchical and multidimensional scaling (MDS) clustering plots associated with the episignature. The result is a combination of the three assessed parameters: MVP scores, hierarchical plots and MDS plots. The result is reported with a confidence level relative to the reference episignature cohoRSTS, where high confidence indicates agreement among all three parameters and moderate confidence indicates disagreement in at least one of the three parameters. In addition to evaluating episignatures, the EpiSign assay also examines the average beta values across a set of previously reported differentially methylated regions.^18,19^ The average beta values are compared to those of unaffected control cohoRSTS, and any value outside of the 1st to 99th percentile range of controls are flagged for further review against cohoRSTS of positive cases.

*Methylation Sensitive Multiplex Ligation-dependent Probe Amplification (MS_MLPA)*

We analysed methylation profile of four differentially methylated regions (DMR) in the GNAS locus using the MS_MLPA assay (ME031-C1 GNAS; MRC Holland). This probemix was also used to detect deletions/duplications in the GNAS complex locus and the STX16 gene.

The GNAS locus is a complex imprinted locus on chromosome 20 that generates multiple transcripts through the use of several alternative first exons that splice into a common set of downstream exons (see Figure 1S). GNAS itself encodes the Gsα protein, which is the α-subunit in the heterotrimeric G protein. Due to differential methylation of their promoters, most gene products originate from one parental allele. Transcripts GNASXL, which encodes XLαs, GNAS A/B (also referred to as 1A) and the antisense transcript GNAS-AS1 (also referred to as NESPAS) are transcribed from the paternal allele, while NESP55 (also referred to as NESP or GNAS transcript variant 4) is transcribed from the maternal allele.^12^ The most downstream promoter (GNAS exon 1) is not differentially methylated, which results in GNAS expression from both alleles in most tissues, but its expression is silenced from the paternal allele in a small number of tissues.^12^

The Gsα and XLαs transcripts are involved in downstream signalling from parathyroid hormone (PTH), parathyroid hormone related protein (PTHrP) receptors and other hormone receptors like TSHR and GHRHR. The GNAS A/B transcript and the antisense transcript GNAS-AS1 are not translated into proteins but are thought to influence Gsα expression via mechanisms that remain to be determined. The STX16 gene, lastly, is a long-range control element of methylation at the GNAS locus, located more than 220 kb centromeric of GNAS.^12^

PHPIb is caused by loss-of-methylation (LOM) at GNAS A/B located within DMR 1 in the GNAS complex locus (Figure 1). LOM can also be observed at GNAS-AS1 and GNASXL, which can also be associated with a gain-of-methylation (GOM) at NESP55. The autosomal dominant form of PHPIb can be caused by maternal heterozygous deletions in STX16. ^12^

More information is available at <https://www.ncbi.nlm.nih.gov/books/NBK459117/>

**RESULTS**

*Clinical description of subject #1*

Subject #1 was a 9-year-old female, born at 40 weeks of gestation via spontaneous vaginal delivery as the second child to non-consanguineous parents of European descent. The pregnancy was largely uncomplicated, except for a report of polyhydramnios, and prenatal ultrasounds were normal. The family history was unremarkable, except for a paternal cousin with febrile convulsions and a maternal grandmother with a history of seizures. The Apgar scores were 8 at both 1 and 5 minutes. At birth, her weight was 2,424g (3^rd^ percentile, -1.91 SD), her length 45.5 cm (4^th^ percentile, -1.77 SD), and her occipitofrontal circumference (OFC) was 29.2 cm (<1^st^ percentile, -3.80 SD). Minor facial abnormalities, choanal atresia, transposition of large vessels, bronchodysplasia and pyelectasia with spontaneous resolution were observed at birth. At 11 days old, she underwent successful surgery of criss cross heart (CCH). During the neonatal period, the patient experienced difficulties in breathing and eating, as well as recurrent vomiting. Developmental parameters were poor with autonomous walking achieved at 24 months and the first words spoken at 5-6 years. Currently, she can say a few sentences and has good communication skills. Bilateral mixed hearing loss was observed at 2 and 8 years of age, accompanied by a type E tympanogram.^20^ At the last evaluation at 8 years, her measurements were: weight 24 kg (31st percentile, -0.49 SD), height 117 cm (3rd percentile, -1.89 SD), and OFC 47 cm (microcephaly, <1st percentile, -3.76 SD). Physical examination revealed thin skin with a visible venous reticulum at the chin, a flat angioma on the forehead, a short and broad neck, an asymmetrical chest, and a slightly prominent abdomen. Facial features included a round face, high nasal root, highly arched eyebrow, downslanted and short palpebral fissures, small and abnormal earlobe, microretrognathia and abnormality of buccal rhyme. Dental malposition and lip hypotonia were also observed. Her hands were small, with bilateral clinodactyly of the 5th finger; her feet exhibited bilateral cutaneous syndactyly of the 1st and 2nd toes and generally short toes (Figure 1A). At 8 yrs. Of age, the speech and language evaluation reported that she could use both non-verbal and verbal communication. However, her developmental abilities were delayed, with pre syntactic combinatory skills equivalent to those of a 19–24-month-old child and verbal comprehension at the level of a 30–35-month-old. A visual agenda was suggested to aid her communication. Additionally, she exhibited attention and self-regulation difficulties. Despite these challenges, her sleep-wake rhythms were normal, and her facial and gestural expressions were appropriate. Fine motor skills required support, and she had not yet acquired night sphincter control. She was diagnosed with mild ID characterized by language impairments and immature emotional and affective development. At 8 years old, echocardiography revealed a possible small dilatation of the aortic bulb.

*Clinical description of subject #2*

Subject #2 was a 32-year-old male of Caucasian origin, born to healthy, non-consanguineous parents. His weight was 74 kg (62nd percentile, +0.30 SD), and his height was 165 cm (5th percentile, -1.61 SD). Microcephaly was reported at birth. He was diagnosed with autism spectrum disorder (ASD) and ID and was mostly non-verbal. He also experienced anxiety and seizures. Similar to subject #1, aortic arch anomalies and hearing loss were reported. Multiple congenital anomalies were noted, including a submucous cleft of the hard palate, bilateral inguinal hernias, absent tear ducts, and short stature. Facial dysmorphisms included epicanthus, thick and highly arched eyebrows, up-slanted palpebral fissures, strabismus, a midline defect of the nose, and clinodactyly of the hands (Figure 1A). Other significant medical conditions included episodes of pneumonia, high cholesterol, hypothyroidism, hyperlipidemia, and dysphagia. At the last evaluation, several gastrointestinal issues were reported, including bowel obstruction, gastroesophageal reflux disease (GERD), mild chronic gastritis, and mild chronic inflammation of the esophagus. Papillomatous lesions of the soft palate and multiple gastric polyps in the rectum and anus were also observed. At the age of 32, he passed away due to a colon carcinoid tumor, which had invaded the submucosa. Familial history of late-onset cancer was positive on the maternal side: breast tumors were reported in several female family members (mother, grandmother, great-grandmother, uncle, and cousin), colon cancer in the grandmother, and mesothelioma due to asbestos exposure in the grandfather.

*Genetic analyses*

Subject #1 involved a comprehensive series of genetic tests to elucidate the underlying cause of the patient's condition. Initially, the patient underwent karyotype analysis, FISH for 22q11.2 and 10p14, trisomy testing for chromosomes 13, 18, and 21, and array-CGH. All these tests returned negative results except for a likely benign duplication at 7p22.3 of maternal origin. Given the clinical presentation, CHARGE syndrome (OMIM #214800) was suspected, but analysis of the CHD7 gene did not confirm this diagnosis. Further investigations using parallel sequencing targeting the NIPBL, SMC3, SMC1A, HDAC8, and RAD21 genes did not identify any pathogenic variant(s). Following these negative results, the patient was enrolled in the “NeuroWES” research program aimed at analyzing NDD subjects by trio whole exome sequencing. This analysis identified a de novo splice site variant in the EP300 gene (NM_001429.4:c.3671+5G>C; p.?). This variant was absent from the Genome Aggregation Database (GnomAD ver 4.1.0) and classified as pathogenic according to the ACMG criteria (applied criteria: PM2 strong, PM4 strong and PP3 strong).^21^

Subject #2 underwent karyotype analysis and FISH testing for 22q deletion and Williams-Beuren syndrome, as well as a SNP array, Fragile-X testing, and a CancerNext Expanded panel from Ambry Genetics. All tests for disorders of glycosylation returned normal results. Then, parallel sequencing was employed utilizing a gene panel designed to identify both syndromic and non-syndromic causes of short stature. This analysis revealed a different variant in the EP300 gene involving the same splice site (NM_001429.4:c.3671+5_3671+8delGTAA; p.?). The variant was confirmed as a de novo event, was private (GnomAD ver 4.1.0), and classified as pathogenic according to ACMG criteria (applied criteria: PM2 moderate, PVS1 strong, and PP5 strong).

*Effect of EP300:c.3671+5G>C on exon 20 skipping*

The c.3671+5G>C variant in the EP300 gene, located in intron 20, was predicted to cause the skipping of exon 20. To validate the functional impact of this variant, peripheral blood mononuclear cells (PBMCs) from the affected individual #1 were analyzed. Amplification of cDNA using specific designed to amplify a coding stretch encompassing exons 20 and 21 revealed the occurrence of an aberrant transcript processing resulting in an in-frame deletion of 81 bp (entire exon 20) (Figure 1B). While biological material was not available for experimentally validating the functional impact of the second variant, c.3671+5_3671+8delGTAA, bioinformatics predictions predicted exon 20 skipping (MaxEnt: -37.4%; NNSPLICE: -72.1%; SSF: -14.7%) (Figure 1C). To understand the impact of the loss of exon 20 on the EP300 protein, an in silico model was generated. This model demonstrated a significant alteration in the three-dimensional structure with a partial loss of the RING_CBP-p300 domain (Figure 1D).

*DNAm analysis in subject #1*

The DNAm analysis also unexpectedly identified hypomethylation at the maternal GNAS locus (OMIM *139320) detailed in supplement file and figure 1S-2S. This finding led us to re-evaluate the exome data, but we did not find any additional causative variants. Loss of methylation in the maternal allele of the imprinted GNAS complex locus is associated with pseudohypoparathyroidism type 1B (OMIM #603233), which is characterized by elevated serum parathyroid hormone (PTH), and impaired levels of calcium and phosphate. Biochemical exams for subject#1 showed normal levels of calcium, phosphate, TSH, and 25-OHvitD (42.6 ng/ml, range 30-100) but slightly increased levels of LDH (293 U/L, range 120-250), CK (202 U/L, range 30-145), and PTH (38.2 pg/mL, range 6.5-36.8). Using the MS_MLPA analysis, we confirmed the hypomethylation at the A/B DMR of the GNAS locus, but were unable to identify a definitive molecular cause, because no deletion was present at GNAS/STX16 loci. (Supplementary file for details).^22,23^


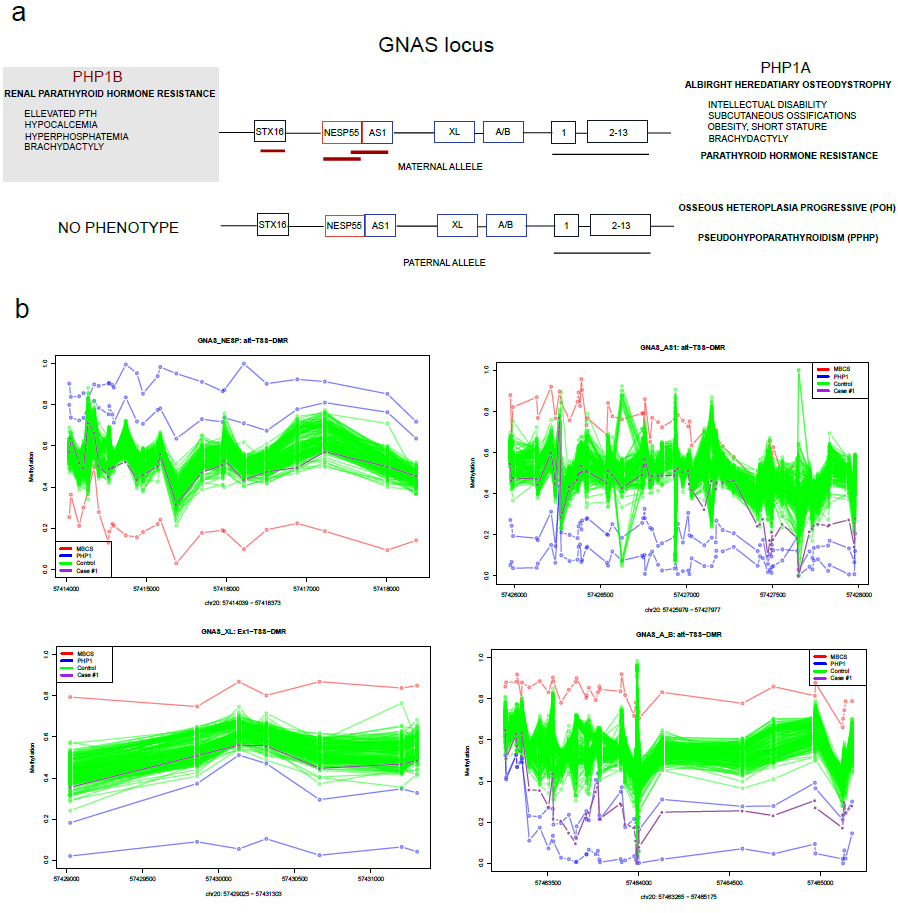


**Figure 1S**. GNAS complex locus and DMR of the GNAS complex locus in case 1. A. Schematic representation of GNAS complex locus (OMIM * 139320) and the associated disease. PHP1B (OMIM * 603233) is defines as defined as isolated resistance to PTH in the absence of AHO. The most frequent cause is an isolated imprinting defect at GNAS A/B:TSS-DMR (A/B) secondary of a 3.0 kb deletion at the maternal allele affecting *STX16;^24^ NESP55*; small deletions at exons 3; exon 4 and intron 3 or intron 4 of GNAS-AS1.^25^ When the deletion is on the paternal allele no phenotype is present. PHP1A (OMIM * 103580) is caused by point mutations and gene rearrangements within the maternal allele of exons 1-13 of the GNAS gene. PPHP (OMIM * 612463) or POH (OMIM * 166350) are the associated diseases when that deletion is affecting the paternal allele. (panel a-orange box-methylated paternally; blue boxes-methylated maternally; red lines-deletions affecting STX16, NESP55 and AS1). In panel B plots representing the DMR of the GNAS complex locus (GNAS- NESP:TSS- DMR; GNAS_AS1:TSS- DMR, GNAS- XL:Ex1-DMR and GNAS A/B:TSS-DMR); red line- Mulchandani-Bhoj-Conlin Syndrome (MBCS); blue line-PHP1; green line-controls; purple line-case 1#. The causative genetic variation for the LOM of GNAS A/B:TSS DMR has still not been detected. Primary epimutations could be one possible explanation.^25^ Legend: PTH-parathyroid hormone; AHO-Albright Hereditary Osteodysrtopy; LOM-loss of methylation.


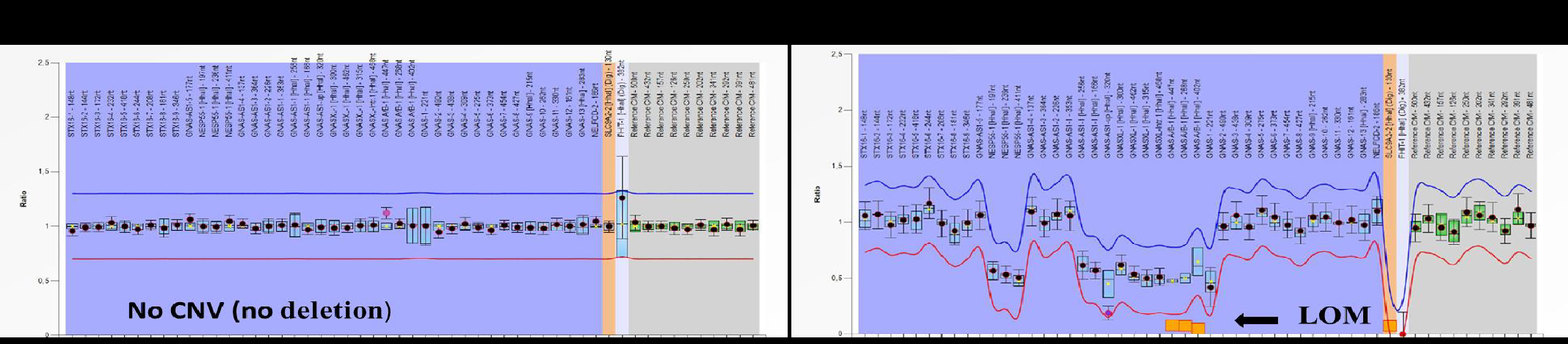


**Figure 2S.** Representative plots of the MS-MLPA analysis by Coffalyzer illustrating the loss of methylation (LOM) at the *GNAS* locus exon A/B in patient #1. Left panel: copy number analysis. The data indicate a normal biallelic status of the *STX16* and *GNAS* region, suggesting the absence of structural defects. Right panel. Methylation analysis data show a loss of imprinting at the *GNAS* exon A/B. The Y-axis represents the methylation ratios, while the X-axis corresponds to the various probes used. Orange squares indicate the region where a loss of methylation is observed.

The autosomal dominant form of PHPIb can be caused by maternal heterozygous deletions in *STX16*, which is not present in patient #1.^12^ The origin of the LOM is therefore unknown.


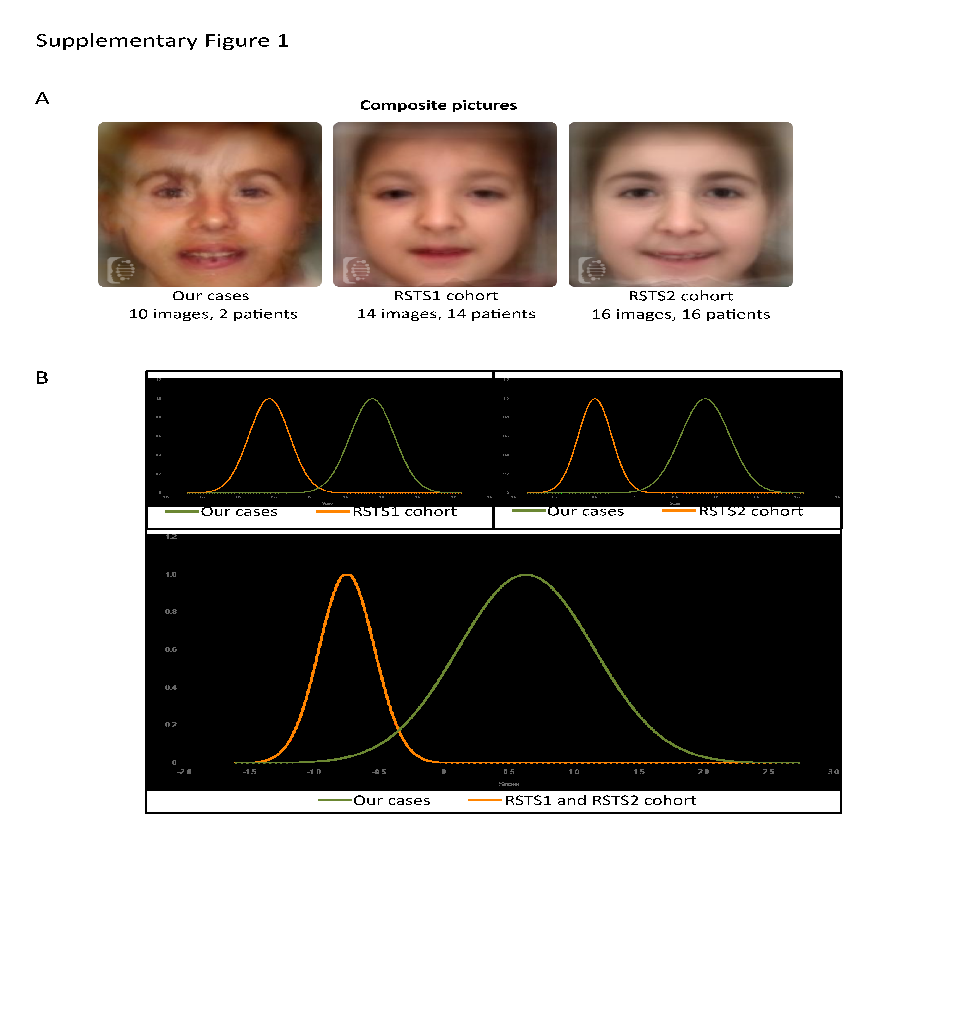


**Figure 3S.** Deep face gestalt analysis performed using Face2Gene web tool (<https://www.face2gene.com/>). For the analysis, our patients’ pictures were compared with 14 images of previously published RSTS1 cases and 16 images of previously published RSTS2 cases (panel A). The score distribution (panel B) shows no overlap between our cases and the other two cohorts, neither when compared alone (upper graphs of panel B) or in combination (lower graphs of panel B).

**Table 1S**

| ***Clinical features observed in RSTS2 patients described in the literature**** | | | | ***Our cohort* (n= 2)** | |
| --- | --- | --- | --- | --- | --- |
|  |  | **Total number (n= 89)** | **Percentage** | **Patient #1** | **Patient #2** |
| ***Behavioral psychiatric manifestations*** | Anxiety | 10/77 | **13%** |  | **+** |
|  | Aggressiveness | 4/64 | **6%** |  |  |
| ***Neurodevelopmental issues*** | ADHD | 3/20 | **15%** | **+** |  |
|  | Autism spectrum disorder (ASD) | 20/80 | **25%** |  | **+** |
|  | Motor delay | 49/66 | **74%** | **+** |  |
|  | Developmental delay | 9/18 | **50%** | **+** |  |
|  | White matter anomalies | 1/12 | **8%** |  |  |
|  | Spina bifida | 2/64 | **3%** |  |  |
|  | Epilepsy | 5/65 | **8%** |  | **+** |
|  | Hearing loss difficulties | 2/27 | **7%** | **+** | **+** |
|  | Hypotonia | 2/12 | **17%** |  |  |
|  | Mild intellectual disability (ID) | 35/65 | **54%** | **+** |  |
|  | Moderate intellectual disability (ID) | 24/65 | **37%** |  |  |
|  | Severe intellectual disability (ID) | 6/65 | **9%** |  | **+** |
|  | Encephalocele/meningocele | 2/54 | **4%** |  |  |
|  | Sleep disturbances | 6/66 | **9%** |  |  |
|  | Speech delay | 43/71 | **61%** | **+** | **+** |
| ***Craniofacial features*** | Malar flattening | 1/12 | **8%** |  |  |
|  | Microcephaly | 77/89 | **87%** | **+** | **+** |
|  | Brachycephaly | 1/12 | **8%** |  |  |
|  | Downslanted palpebral fissures | 45/83 | **54%** | **+** |  |
|  | Highly arched eyebrows | 51/82 | **62%** | **+** | **+** |
|  | Long eyelashes | 63/76 | **83%** |  |  |
|  | Epicanthal folds | 13/72 | **18%** |  | **+** |
|  | Low set ears | 18/74 | **24%** |  |  |
|  | Rotated ears | 6/26 | **23%** |  |  |
|  | Abnormal helix | 9/69 | **13%** |  |  |
|  | Abnormal earlobe | 1/12 | **8%** | **+** |  |
|  | Microretrognathia | 35/88 | **40%** | **+** |  |
|  | Pointed chin | 2/12 | **17%** |  |  |
|  | Dental malposition/malocclusion | 6/22 | **27%** | **+** |  |
|  | Narrow palate | 8/26 | **31%** |  |  |
|  | Thin upper lip vermillion | 5/12 | **42%** | **+** |  |
|  | Smooth/short philtrum | 2/12 | **17%** |  |  |
|  | Highly arched palate | 35/65 | **54%** |  |  |
|  | Columella below the alae nasi | 75/89 | **84%** |  |  |
|  | Grimacing smile | 31/73 | **42%** |  |  |
|  | Downturned corner mouth | 2/12 | **17%** |  |  |
|  | Prominent/bulbous nose | 11/25 | **44%** |  |  |
|  | Duplication of distal first phalanx | 1/32 | **3%** |  |  |
|  | Brachytelephalangy | 7/32 | **22%** |  |  |
|  | Anteverted nares | 3/12 | **25%** |  |  |
|  | Prominent nasal bridge | 4/18 | **22%** |  |  |
|  | Narrow nose | 1/12 | **8%** |  |  |
|  | Wide nasal bridge | 2/12 | **17%** |  |  |
|  | Convex nasal bridge | 26/66 | **39%** |  |  |
| ***Dermatological features*** | Hirsutism | 9/25 | **36%** |  |  |
|  | Hypertrichosis | 29/59 | **49%** |  |  |
|  | Low implantation hairline | 33/71 | **46%** |  |  |
|  | Pilomatricomas | 7/51 | **14%** |  |  |
|  | Keloids | 6/77 | **8%** |  |  |
|  | Eczema | 3/12 | **25%** |  |  |
| ***Musculoskeletal features*** | Ankle contractures | 2/12 | **17%** |  |  |
|  | Spinal anomalies | 3/14 | **21%** |  |  |
|  | Short stature | 2/14 | **14%** |  | **+** |
|  | Scoliosis | 16/80 | **20%** |  |  |
|  | Small hands | 3/12 | **25%** | **+** |  |
|  | Short feet | 3/12 | **25%** | **+** |  |
|  | Long fingers | 2/64 | **3%** |  |  |
|  | Long toes | 1/12 | **8%** |  |  |
|  | Broad great toes | 1/12 | **8%** |  |  |
|  | Broad distal phalanges | 4/64 | **6%** |  |  |
|  | Broad fingertips | 25/57 | **44%** |  |  |
|  | Flat feet | 4/21 | **19%** |  |  |
|  | Cutaneous syndactyly of toes | 3/64 | **5%** |  |  |
|  | Bilateral syndactyly of fingers | 5/73 | **7%** |  |  |
|  | Radial deviation of the hands | 3/70 | **4%** |  |  |
|  | Talon cusps | 3/58 | **5%** |  |  |
|  | Broad thumbs | 62/89 | **70%** |  |  |
|  | Pectus excavatum | 7/66 | **11%** |  |  |
|  | Broad halluces | 63/88 | **72%** |  |  |
|  | Angulated thumbs | 4/69 | **6%** |  |  |
| ***Metabolic, ophtalmologic and other systemic features*** | Cardiovascular anomalies | 24/75 | **32%** | **+** | **+** |
|  | Upper respiratory tract infections | 1/18 | **6%** |  |  |
|  | Respiratory anomalies | 5/21 | **24%** | **+** | **+** |
|  | Urinary tract anomalies | 17/73 | **23%** |  |  |
|  | Cryptorchidism | 4/35 | **11%** |  |  |
|  | Recurrent genitourinary infections | 2/64 | **3%** |  |  |
|  | Feeding problems | 9/33 | **27%** | **+** | **+** |
|  | Obesity | 20/66 | **30%** |  |  |
|  | Constipation | 5/14 | **36%** |  |  |
|  | Gastric reflux | 11/72 | **15%** |  | **+** |
|  | Myopia | 14/79 | **18%** |  |  |
|  | Synophrys | 2/12 | **17%** |  |  |
|  | Astigmatism | 4/68 | **6%** |  |  |
|  | Hyperopia | 2/62 | **3%** |  |  |
|  | Hypermetropia | 4/23 | **17%** |  |  |
|  | Lacrimal duct anomalies | 1/54 | **2%** | **+** | **+** |
|  | Strabismus | 24/80 | **30%** |  | **+** |
|  | Hypoglycemia | 3/20 | **15%** |  |  |
|  | Anemia (including hypoplastic) | 2/64 | **3%** |  |  |
|  | Hypokalemia | 1/52 | **2%** |  |  |
|  | Immunologic anomalies | 2/21 | **10%** |  |  |
|  | Polyhydramnios | 2/66 | **3%** | **+** |  |
|  | Oligohydramnios | 1/52 | **2%** |  |  |
|  | Postnatal growth retardation | 47/78 | **60%** |  |  |
|  | Prenatal growth retardation | 31/73 | **42%** |  |  |
|  | Preeclampsia | 20/89 | **22%** |  |  |
|  | Cancer | 3/70 | **4%** |  | **+** |
|  |  |  |  |  |  |
| ** Cohen et al., 2020 ^26^ (12 patients), Fergelot et al., 2016 ^27^ (52 patients), Enomoto et al., 2022^28^ (2 patients), López et al., 2018 ^29^ (8 patients), Negri et al., 2015 ^30^ (6 patients), Hamilton et al. 2016 ^31^ (9 patients)*  **Table 1S.** The table presents a detailed summary of clinical features observed in RSTS2 patients described in the literature with those in our cohort (n=2). The literature data is aggregated from multiple sources, including *^26-31^*, for a total of 89 patients. For each clinical feature listed, the total number and percentage of affected patients in the literature are provided alongside the presence (+) of the feature in Patient #1 and Patient #2 from our cohort. This comparative analysis highlights the ratios and distribution of concerned clinical manifestations, offering valuable insights into the variability and general occurrence of symptoms among different patient groups and studies. | | | | | |

**Table 2S**

| ***Additional clinical and phenotypic features in our cases*** | |  |  |
| --- | --- | --- | --- |
|  |  | **Case #1** | **Case #2** |
| ***Craniofacial features*** | Round face | + |  |
|  | Upslanting palpebral fissures |  | + |
|  | Broad neck | + |  |
|  | Short neck | + |  |
|  | Deviation of buccal rhyme | + |  |
|  | High root of nose | + |  |
|  | Deviation of nasal septum |  | + |
|  | Submucous cleft of the hard palate |  | + |
|  | Choanal atresia | + |  |
| ***Dermatological features*** | Angioma | + |  |
|  | Thin skin | + |  |
| ***Musculoskeletal features*** | Asymmetrical chest | + |  |
|  | Prominent abdomen | + |  |
|  | Clinodactyly | + | + |
| ***Metabolic, ophthalmologic and other systemic features*** | High cholesterol |  | + |
|  | Hypothyroidism |  | + |
|  | Hyperlipidemia |  | + |
|  | Pyelectasia | + |  |
|  | Bilateral inguinal hernias |  | + |
|  | Bronchodysplasia | + |  |

**Table 2S.** This table highlights the additional clinical and phenotypic features observed in our cases (n=2). The features are categorized into craniofacial, dermatological, musculoskeletal, and metabolic, ophthalmologic, and other systemic features. The presence (+) of each feature in Case #1 and Case #2 is indicated. The table provides a clear comparison of the specific characteristics noted in our patients, contributing to a deeper understanding of the phenotypic spectrum associated with Rubinstein-Taybi Syndrome (RSTS).

**REFERENCES**

1. Bauer CK, Calligari P, Radio FC, et al. Mutations in KCNK4 that Affect Gating Cause a Recognizable Neurodevelopmental Syndrome. *Am J Hum Genet.* 2018;103(4):621-630.

2. De Rubeis S, He X, Goldberg AP, et al. Synaptic, transcriptional and chromatin genes disrupted in autism. *Nature.* 2014;515(7526):209-215.

3. Satterstrom FK, Kosmicki JA, Wang J, et al. Large-Scale Exome Sequencing Study Implicates Both Developmental and Functional Changes in the Neurobiology of Autism. *Cell.* 2020;180(3):568-584.e523.

4. Flex E, Martinelli S, Van Dijck A, et al. Aberrant Function of the C-Terminal Tail of HIST1H1E Accelerates Cellular Senescence and Causes Premature Aging. *Am J Hum Genet.* 2019;105(3):493-508.

5. Van der Auwera GA, Carneiro MO, Hartl C, et al. From FastQ data to high confidence variant calls: the Genome Analysis Toolkit best practices pipeline. *Curr Protoc Bioinformatics.* 2013;43:11 10 11-33.

6. Li H, Durbin R. Fast and accurate short read alignment with Burrows-Wheeler transform. *Bioinformatics.* 2009;25(14):1754-1760.

7. Cingolani P, Platts A, Wang le L, et al. A program for annotating and predicting the effects of single nucleotide polymorphisms, SnpEff: SNPs in the genome of Drosophila melanogaster strain w1118; iso-2; iso-3. *Fly (Austin).* 2012;6(2):80-92.

8. Liu X, Wu C, Li C, Boerwinkle E. dbNSFP v3.0: A One-Stop Database of Functional Predictions and Annotations for Human Nonsynonymous and Splice-Site SNVs. *Hum Mutat.* 2016;37(3):235-241.

9. Kircher M, Witten DM, Jain P, O'Roak BJ, Cooper GM, Shendure J. A general framework for estimating the relative pathogenicity of human genetic variants. *Nat Genet.* 2014;46(3):310-315.

10. Jagadeesh KA, Wenger AM, Berger MJ, et al. M-CAP eliminates a majority of variants of uncertain significance in clinical exomes at high sensitivity. *Nat Genet.* 2016;48(12):1581-1586.

11. Li Q, Wang K. InterVar: Clinical Interpretation of Genetic Variants by the 2015 ACMG-AMP Guidelines. *Am J Hum Genet.* 2017;100(2):267-280.

12. Turan S, Bastepe M. The GNAS complex locus and human diseases associated with loss-of-function mutations or epimutations within this imprinted gene. *Horm Res Paediatr.* 2013;80(4):229-241.

13. Robinson JT, Thorvaldsdóttir H, Winckler W, et al. Integrative genomics viewer. *Nat Biotechnol.* 2011;29(1):24-26.

14. Kopanos C, Tsiolkas V, Kouris A, et al. VarSome: the human genomic variant search engine. *Bioinformatics.* 2019;35(11):1978-1980.

15. Kerkhof J, Squeo GM, McConkey H, et al. DNA methylation episignature testing improves molecular diagnosis of Mendelian chromatinopathies. *Genet Med.* 2022;24(1):51-60.

16. Levy MA, Relator R, McConkey H, et al. Functional correlation of genome-wide DNA methylation profiles in genetic neurodevelopmental disorders. *Hum Mutat.* 2022;43(11):1609-1628.

17. Levy MA, McConkey H, Kerkhof J, et al. Novel diagnostic DNA methylation episignatures expand and refine the epigenetic landscapes of Mendelian disorders. *HGG Adv.* 2022;3(1):100075.

18. Kerkhof J, Rastin C, Levy MA, et al. Diagnostic utility and reporting recommendations for clinical DNA methylation episignature testing in genetically undiagnosed rare diseases. *Genet Med.* 2024;26(5):101075.

19. Monk D, Morales J, den Dunnen JT, et al. Recommendations for a nomenclature system for reporting methylation aberrations in imprinted domains. *Epigenetics.* 2018;13(2):117-121.

20. Lidén G, Harford E, Hallén O. Tympanometry for the diagnosis of ossicular disruption. *Arch Otolaryngol.* 1974;99(1):23-29.

21. Richards S, Aziz N, Bale S, et al. Standards and guidelines for the interpretation of sequence variants: a joint consensus recommendation of the American College of Medical Genetics and Genomics and the Association for Molecular Pathology. *Genet Med.* 2015;17(5):405-424.

22. Mantovani G, Spada A, Elli FM. Pseudohypoparathyroidism and Gsα-cAMP-linked disorders: current view and open issues. *Nat Rev Endocrinol.* 2016;12(6):347-356.

23. Thiele S, Mantovani G, Barlier A, et al. From pseudohypoparathyroidism to inactivating PTH/PTHrP signalling disorder (iPPSD), a novel classification proposed by the EuroPHP network. *Eur J Endocrinol.* 2016;175(6):P1-P17.

24. Hanna P, Francou B, Delemer B, Jüppner H, Linglart A. A Novel Familial PHP1B Variant With Incomplete Loss of Methylation at GNAS-A/B and Enhanced Methylation at GNAS-AS2. *J Clin Endocrinol Metab.* 2021;106(9):2779-2787.

25. Mantovani G, Bastepe M, Monk D, et al. Diagnosis and management of pseudohypoparathyroidism and related disorders: first international Consensus Statement. *Nat Rev Endocrinol.* 2018;14(8):476-500.

26. Cohen JL, Schrier Vergano SA, Mazzola S, et al. EP300-related Rubinstein-Taybi syndrome: Highlighted rare phenotypic findings and a genotype-phenotype meta-analysis of 74 patients. *Am J Med Genet A.* 2020;182(12):2926-2938.

27. Fergelot P, Van Belzen M, Van Gils J, et al. Phenotype and genotype in 52 patients with Rubinstein-Taybi syndrome caused by EP300 mutations. *Am J Med Genet A.* 2016;170(12):3069-3082.

28. Enomoto Y, Yokoi T, Tsurusaki Y, et al. Divergent variant patterns among 19 patients with Rubinstein-Taybi syndrome uncovered by comprehensive genetic analysis including whole genome sequencing. *Clin Genet.* 2022;101(3):335-345.

29. López M, García-Oguiza A, Armstrong J, et al. Rubinstein-Taybi 2 associated to novel EP300 mutations: deepening the clinical and genetic spectrum. *BMC Med Genet.* 2018;19(1):36.

30. Negri G, Milani D, Colapietro P, et al. Clinical and molecular characterization of Rubinstein-Taybi syndrome patients carrying distinct novel mutations of the EP300 gene. *Clin Genet.* 2015;87(2):148-154.

31. Hamilton MJ, Newbury-Ecob R, Holder-Espinasse M, et al. Rubinstein-Taybi syndrome type 2: report of nine new cases that extend the phenotypic and genotypic spectrum. *Clin Dysmorphol.* 2016;25(4):135-145.
